# Supplementary material for: Association of body size distortion with low body mass index in female patients with nontuberculous mycobacterial lung disease
Source: PLoS One. 2023 Aug 22;18(8):e0290277. doi: 10.1371/journal.pone.0290277 (PMC10443841; doi:10.1371/journal.pone.0290277)
Supplement: S1 Table — (DOCX) [file pone.0290277.s001.docx]

**S1 Table.** Anthropometric profiles BSP in NTM-LD and Controls with excluding patients BMI less than 16.2 kg/m^2^

|  | NTM-LD  (*n* = 71) | Controls  (*n* = 111) | *p* value |
| --- | --- | --- | --- |
| Age (years) ^a^  Height (cm) ^a^  Body weight (kg) ^a^  Body mass index (kg/m^2^) ^a^  BSP ^b^  Underestimation of body size  No distortion  Overestimation of body size | 70.5 ± 8.9  153.8 ± 6.8  48.2 ± 6.4  55.8 ± 8.5  30 (42.3)  17 (23.9)  24 (33.8) | 57.1 ± 5.2  157.9 ± 5.3  55.8 ± 8.5  22.4 ± 3.2  44 (39.6)  36 (32.4)  31 (27.9) | **<0.001**  **<0.001**  **<0.001**  **<0.001**  0.441 |

Mean ± standard deviation or *n* (%), ^a^ Unpaired t-test, ^b^ *χ^2^* test

BSP, body size perception; NTM-LD, nontuberculous mycobacterial lung disease

Patients were divided into underestimation of body size, no distortion, and overestimation of body size based on the difference between their perceived and actual silhouettes.
