# Supplementary material for: Association of body size distortion with low body mass index in female patients with nontuberculous mycobacterial lung disease
Source: PLoS One. 2023 Aug 22;18(8):e0290277. doi: 10.1371/journal.pone.0290277 (PMC10443841; doi:10.1371/journal.pone.0290277)
Supplement: S2 Table — (DOCX) [file pone.0290277.s002.docx]

**S2 Table.** Comparison of anthropometric and nutritional characteristics between the patient groups with excluding BMI less than 16.2 kg/m^2^

|  | OE  (*n* = 24) | Non-OE  (*n* = 47) | *p* value |
| --- | --- | --- | --- |
| Anthropometric data  Height (cm)  Body weight (kg)  Body mass index (kg/m^2^)  Fat mass (kg)  Percentage of body fat (%)  Skeletal muscle mass (kg)  Weight loss from age 20 (kg)  Percentage of weight loss from age 20 (%)  Weight loss from maximum body weight (kg)  Percentage of weight loss from maximum body weight (%)  Nutritional intake  Energy (kcal/day)  Energy (kcal/IBW1kg/day) ^a^  Protein (g/day)  Protein (g/IBW1kg/day) ^a^  Fat (g/day)  Fat (g/IBW1kg/day) ^a^  Carbohydrate (g/day)  Carbohydrate (g/IBW1kg/day) ^a^  Appetite score (SNAQ-J)  Amount of physical activity (MET·hour/week)  Duration of sitting time (hour/day)  Blood biochemistry  Albumin (g/dL)  Transthyretin (mg/dL) ^b^  Transferrin (mg/dL)  Retinol-binding protein (mg/dL) ^b^  Total cholesterol (mg/dL)  Hemoglobin (g/dL)  Lymphocyte count (×10^2^/μL) | 157.7 ± 6.6  46.2 ± 4.9  19.0 ± 1.7  11.4 ± 3.9  24.2 ± 6.5  18.2 ± 1.9  3.5 ± 6.9  6.4 ± 12.7  8.6 ± 6.6  15.2 ± 10.6  1658 ± 323  31.1 ± 6.4  61.6 ± 18.4  1.2 ± 0.4  49.0 ± 16.5  0.9 ± 0.3  231.5 ± 49.8  4.3 ± 0.9  14.9 ± 1.9  26.8 ± 34.6  4.6 ± 2.9  4.4 ± 0.3  19.4 ± 4.7  219.3 ± 45.9  2.4 ± 0.6  224.3 ± 29.2  13.0 ± 0.8  12.8 ± 4.7 | 152.8 ± 6.7  49.3 ± 6.8  21.0 ± 2.0  14.9 ± 4.1  29.9 ± 5.6  18.0 ± 2.5  -0.6 ± 6.5  -2.0 ± 13.7  6.1 ± 5.5  10.7 ± 8.4  1728 ± 352  33.6 ± 6.3  68.3 ± 17.6  1.3 ± 0.3  54.1 ± 18.0  1.0 ± 0.3  233.8 ± 61.0  4.6 ± 1.1  14.5 ± 1.6  30.9 ± 42.5  4.9 ± 2.0  4.4 ± 0.3  20.4 ± 4.3  224.8 ± 34.1  2.6 ± 0.5  226.5 ± 38.1  13.2 ± 1.3  14.3 ± 3.6 | 0.089  **0.034**  **<0.001**  **0.001**  **<0.001**  0.685  **0.015**  **0.017**  0.094  0.055  0.417  0.126  0.138  0.051  0.253  0.146  0.869  0.428  0.267  0.686  0.627  0.543  0.348  0.568  0.257  0.805  0.675  0.136 |

Mean ± standard deviation, Unpaired t-test (OE vs. Non-OE).

^a^ Normalized using ideal body weight (BMI = 22 kg/m^2^).

^b^ Data (transthyretin and retinol-binding protein) of one patient in the Non-OE group could not be obtained.

NTM-LD, nontuberculous mycobacterial lung disease; IBW, ideal body weight; SNAQ-J, Japanese version of the Simplified Nutritional Appetite Questionnaire
