# Supplementary material for: Association of body size distortion with low body mass index in female patients with nontuberculous mycobacterial lung disease
Source: PLoS One. 2023 Aug 22;18(8):e0290277. doi: 10.1371/journal.pone.0290277 (PMC10443841; doi:10.1371/journal.pone.0290277)
Supplement: S3 Table — (DOCX) [file pone.0290277.s003.docx]

**S3 Table.** Association of BSP with BMI in NTM-LD patients with excluding BMI less than 16.2 kg/m^2^

| Variables ^a^ | *ꞵ* | 95% CI | VIF | p-value |
| --- | --- | --- | --- | --- |
| Body size distortion ^b^  Age (years)  Duration of NTM-LD (month)  Percentage of weight loss from age 20 (%)  Energy intake (kcal/IBW1kg/day)  Appetite score (SNAQ-J)  Amount of physical activity (MET·hour /week) | -0.372  0.127  -0.220  -0.488  0.115  0.090  0.182 | -0.761 – -0.235  -0.018 – 0.078  -0.015 – -0.001  -0.101 – 0.046  -0.026 – 0.101  -0.127 – 0.349  -0.001 – 0.020 | 1.202  1.254  1.026  1.035  1.180  1.152  1.239 | **<0.001**  0.212  **0.018**  **<0.001**  0.244  0.354  0.073 |

*n* = 71. BSP, body size perception; BMI, body mass index; NTM-LD, nontuberculous mycobacterial lung disease; CI, confidence interval; VIF, variance inflation factor; IBW, ideal body weight; SNAQ-J, Japanese version of the Simplified Nutritional Appetite Questionnaire

BMI as the dependent variable, ^a^ Independent variables

^b^ Difference between perceived silhouette and the actual silhouette.
