# Supplementary material for: Association of body size distortion with low body mass index in female patients with nontuberculous mycobacterial lung disease
Source: PLoS One. 2023 Aug 22;18(8):e0290277. doi: 10.1371/journal.pone.0290277 (PMC10443841; doi:10.1371/journal.pone.0290277)
Supplement: Attachment — Submitted filename: Response to reviewers.docx [file pone.0290277.s004.docx]

**Point-by-point response to the Editor and Reviewers**

We are grateful to the editor and reviewers for important comments and useful suggestions. As indicated in the following responses, we have taken all these comments and suggestions into account in the revised version of our manuscript.

**Comments by the Editor**

**Major comments**

**1) Abstract (Methods): Important descriptions on the study design (cross-sectional), how to recruit control women (web survey), and the main statistical analysis (multiple regression analysis) are all missing in the Abstract. Please be explicit about what is main independent variable and a dependent variable in the multiple regression analysis.**

**Answer:** Thank you for pointing this out. We have added descriptions of the study design (cross-sectional), the method of recruitment of control women (web survey), and the main statistical analysis (multivariate regression analysis with one dependent variable) to the revised abstract (line 38-45). The independent variables (body size perceptions, nutritional factors, and physical activity) are not reported in the Methods because they overlap with the revised purpose of the Abstract (line 34-36).

The Methods section of main text also reflects this modification (line 102-112 and 182-185).

**2) Introduction: Overall (not only the Introduction section but also the other sections), the manuscript is written in poor English. The manuscript should be checked by a native speaker.**

**Answer:** Thank you for your comments on the quality of the English text. Actually, the first draft was reviewed by a native speaker of a proofreading company. The revised manuscript was proofread by two native English speakers of another proofreading company.

**3) Line 112: Height and weight in control women are self-reported (not objectively measured). Thus, BMI data in the control group are based on self-reported height and weight. Please be explicit about that point throughout the manuscript.**

**Answer:** Following your comment, it has been added in the revised Abstract and Methods section (line 40 and line 110) that the height and weight of the controls were self-reported. The use of self-reported height and weight was already reported in the limitations of the original manuscript, so no further additions were made.

**4) Lines 156-159: There is almost no information on the questionnaires used in the present study. Citing your previous study is insufficient, brief explanations on the questionnaires are needed. For instance, which questionnaire was used to assess dietary intake? How did you calculate energy, protein, fat, carbohydrate intakes. Is the diet questionnaire used in the present study validated? Did you use validated questionnaires for assessing physical activity and appetite score? What about smoking history?**

**Answer:** We apologize for the lack of information in the Methods section. Following your comments, brief explanations have been added in the revised manuscript (line 145-162). The questionnaires we used have all been validated. Note that the smoking history was obtained from the patients' medical records (line 145-146).

**5) Line 174: In the footnotes of Tables 1-3, it is described that UNPAIRED t-test was used. However, you mentioned paired Student’s t-test or Welch's t-test in this line.**

**Answer:** Thank you for pointing out the discrepancy in the description of the statistical methods.

We have corrected the description of the statistical methods in the footnote to match the description of the methods in the text of Table 1-3.

**6) Line 176: Please be explicit about which variable (BMI or body size distortion) is an independent variable or a dependent variable. What is the purpose of this analysis? Which hypothesis is tested by performing the univariate analysis.? Please explain courteously the reason why you performed this analysis. Or consider leaving it out. I can understand if you use body size distortion as an independent variable (since the body size distortion is a potential cause) and BMI as a dependent variable (since BMI is an outcome), as you did in the followed multiple regression analysis (as shown in Table 4) in order to examine your initial hypothesis described in the Introduction section. However, I could not clearly understand your intent for performing the univariate analysis, in which BMI is treated as an independent variable, and body size distortion is treated as a dependent variable.**

**Answer:** We appreciate your very important suggestions. Our study began with the question: "Why do female patients with NTM not eat enough even though they are thin? Our hypothesis was that "in female patients with NTM, body size distortion (overestimation) leads to low BMI". Thus, BMI is the dependent (objective) variable and body size distortion is the independent variable. However, the original manuscript was confusing to the reader due to unclear statements. In the revised manuscript, the following corrections were made in the Title, Abstract, and throughout the text. In addition, the single regression analysis has been removed (Table 4 in the original manuscript). Also, factors (independent variables) influencing BMI were carefully re-checked in the multiple regression analysis (Table 4).

● *Original Title:*

Association of **body size perception** with body mass index, long-term energy balance, and nutritional status in female patients with pulmonary nontuberculous mycobacterial lung disease

↓

● *Revised Title:*

Association of **low body mass index** with body size distortion in female patients with non-tuberculous mycobacterial lung disease

● *Original Abstract (line 34-37):*

“We therefore hypothesized that the patients' reduced appetites were due to a misperception of their body shape. To test this hypothesis, we examined the association of body size perception (BSP) with body mass index (BMI) and nutritional status among the patients.”

↓

● *Revised Abstract (line 33-36):*

“We hypothesized that **their thinness was associated with a misperception of body shape**. To test this hypothesis, we examined **whether the patients’ body mass index (BMI) was related to their body size perception (BSP), independent of age, disease duration, and nutritional factors**.”

● *Original Introduction (line 92-100):*

“Accordingly, we hypothesized that a certain percentage of female patients with NTM-LD have body size distortion similar to those of the general adult female population, and that their overestimation of body size has an influence on their thinness and nutritional status. To test this hypothesis, we 1) compared female patients with NTM-LD with ordinary women in terms of body size perception and distortion, as assessed using a Japanese version of the body image scale we developed in a previous study [19], and 2) examined the impact of body size overestimation on their body size and nutritional status.”

↓

● *Revised Introduction (line 90-98):*

“Accordingly, we hypothesized that some female patients with NTM-LD have body size distortion similar to that of the general adult female population, and that their overestimation of body size would influence their thinness (**low BMI**).

To test this hypothesis, we 1) compared female patients with NTM-LD to healthy controls in terms of body size perception (**BSP**) and distortion, as assessed by the Japanese version of the Body Image Scale developed in a previous study [19], and 2) examined whether **patients' BMI was related to their BSP, independent of disease duration and nutritional factors**.”

**7) Lines 180-184: As Reviewer 1 pointed out, it is critically important that this sentence should be rephrased to “impact of age etc (explanatory or independent variables) on BMI (outcome or dependent variables)”.**

**Answer:** Based on your suggestion, we have made the following correction, which is related to major comment 6).

● *Original Methods (line 180-184):*

“Multiple regression analysis was performed to evaluate the impact of BMI (response variable) on age, duration of NTM-LD, percentage of weight loss from 20 years of age, normalized energy intake, appetite score, amount of physical activity, and body size distortion (explanatory variables).

↓

● *Revised Methods (line 182-185):*

“Multiple regression analysis was performed to evaluate the impact of age, duration of NTM-LD, percentage of weight loss from 20 years of age, normalized energy intake, appetite score, amount of physical activity, and body size distortion (independent variables) on BMI (dependent variable).”

To be consistent with the above changes, the Abstract and the Description of the discussion have been modified as follow:

● *Revised Abstract (line 51-53) and Discussion (line 272-274):*

“Multiple regression analysis revealed that longer disease history, longitudinal weight loss, and overestimation of body size were all independently associated with lower BMI”

●*Revised Conclusion (line 351-354):*

“The present findings suggest that body size distortion is an etiological factor for lower BMI in female patients with NTM-LD. This result supports our hypothesis that patients' thinness is associated with body shape distortion. It may be important to understand patients' body image when providing dietary advice.”

**8) In the Introduction section (lines 98-100), you mentioned that you examined the impact of body size overestimation on their body size and nutritional status. To examine this, you should treat the body size overestimation (potential cause) as an independent variable and the body size (BMI) and nutritional status (potential outcomes) as dependent variables in the association analysis. However, you treat energy intake (nutritional status) as an independent variable in the multiple regression analysis (results show in Table 4). Why?**

**Answer:** Thank you for your question. Our response to this comment is included in our response to **Comment 6**.

**9) You treated the body size distortion as a main independent variable in the multiple regression analysis. Please clearly explain whether you treat other six independent variables (age, duration of NTM-LD, percentage of weight loss from age 20, etc) as potential confounding factors or mediating factors. Usually, mediating factors should not be included as covariates in multivariate regression models, since the inclusion of mediating factors into multivariate models disappears the significant association between a main independent variable and an outcome.**

**Answer:** Thank you very much for your important comments. As mentioned in the response to **Comment 6**, we have revised the hypotheses in the Introduction and the description of the dependent and independent variables in the multiple regression analysis.

The 6 independent variables were confirmed to be non-mediating factors by the following analyses. Based on these results, we believe it is appropriate to treat the six independent variables as potential confounders.

● We performed a mediation analysis using the R mediation package.

<https://imai.fas.harvard.edu/research/files/mediationR2.pdf>

We produced an average causal mediation effect (ACME), an average direct effect (ADE) and total effect using the R mediation package.

Causal mediation analysis showed that weight loss from age 20 years did not mediate -0.146 (95% CI -0.388-0.050) of BSP in BMI (P=0.140 for ACME) (Table and Figure). Also, other factors did not mediate of in BMI.

Table R1. ACME, ADE, and total effect.

|  | Estimate | 95% CI Lower | 95% CI Upper | p-value |
| --- | --- | --- | --- | --- |
| ACME  ADE  Total Effect  Prop. Mediated | -0.1460 -0.5505  -0.6965  0.1986 | -0.3879  -0.8098  -1.0449  -0.0905 | 0.05  -0.29  -0.36  -0.0905 | 0.14  <2e-16 ***  <2e-16 ***  0.14 |

Signif. codes: 0 '***' 0.001 '**' 0.01 '*' 0.05 '.' 0.1 ' ' 1

Figure R1. ACME, ADE, and total effect.

Based on the above, the following corrections have been made:

● The VIF values have been added to Table 4 in the revised main text (line 259).

● The following sentence has been added to the Statistical Analysis in the revised main text (line 185-187).

“We obtained an average causal mediation effect (ACME), an average direct effect, and a total effect using the R mediation package.”

● The following sentence has been added to the Results section in the revised main text (line 253-255).

“Causal mediation analysis showed that weight loss from age 20 years did not mediate −0.146 (95% CI, −0.388 to 0.050) BSP in BMI (p = 0.140 for ACME).”

**10) Lines 267: Throughout the results section, please be explicit about which variable is an independent variable or a dependent variable.**

**Answer:** Following your comment, the independent and dependent variables have been added to the Results section in the revised main text (line 248-249, and 264).

**11) Line 277: Discussion must be appropriately revised according to the changes you made in the Methods and Results sections according to reviewers’ and my comments.**

**Answer:** Following all the comments from the editor and the reviewers, we have modified the Discussion in the revised main text (line 268-348).

**Minor comments**

**1) Line 87: full name first, abbreviation second.**

**Answer:** Based on your comment, the following change has been made:

●*Original (line 87)*

OECD (Organization for Economic Co-operation and Development)

↓

●*Revised (line 85-86)*

Organization for Economic Co-operation and Development (OECD)

**2) Lines 256-258: βvalues are missing.**

**Answer:** Based on your comment, βvalues have been added to the Results (line 250-252) in the revised main text.

**Comments by the Reviewer #1**

**This study is useful in providing greater insight into some reasons underlying the lower BMI among Japanese women with NTM lung disease, namely a distorted body perception, as well as the factors predictive of low BMI in this same population. The study is thorough, and adds important information to the field. In particular, the role of low BMI in disease susceptibility is clear, and the difficult and often complicated factors underlying the low BMI and the need for improved intervention are highlighted in this paper.**

**Answer:** Thank you for taking the time to review our manuscript. We appreciate your positive and constructive feedback.

**Minor comments**

**1) Suggest finding a term other than “elderly”, as this term is subjective; suggest instead explicitly using an age value.**

**Answer:** As you suggested, we have changed to using the age value instead of "elderly”. All "elderly" words have been removed from the revised manuscript.

●*Original Methods (line 112-114):*

“We selected control participants (n=111) who possessed a similar demographic background as the patients with NTM-LD (Japanese, female, mostly **elderly**).”

↓

●*Revised Methods (line 110-112):*

“We selected control participants (n = 111) with a similar demographic background as the patients with NTM-LD (**Japanese females aged 50–75 years**).”

●*Original Discussion (line 297-299):*

“Our NTM-LD patients were thinner than the control women and were in their pre-elderly years, but up to 40% of them overestimated their body size. “

↓

●*Revised Discussion (line 277-286):*

“About 40% of the present patients overestimated their body size, which was higher than the about 30% of control women in the general population. …. The lower incidence of overestimation in the general female population in the present study than in the early studies may be due to the younger age of the participants (> 17 years [27−29] vs. > 50 years), as younger age is more likely to lead to overestimation of body size in adult women [18].“

**2) Find a better term than “ordinary”- suggest “controls”. Please be consistent in referring to “controls” rather than “ordinary”.**

**Answer:** We have changed from “ordinary” to “controls” in the Results section. In addition, we have modified the term "ordinary" throughout the revised manuscript. For example, in the description of previous studies in the Abstract and Introduction, we have changed the term "ordinary women" to "healthy controls".

**3) Results**

**Controls were on average 10 years older than cases… was it not possible to find controls within the same general age range? Ie frequency match?**

**Answer:** The average age of the controls was approximately 13 years younger than that of the NTM-LD patients. We regret that we could not match the ages of the two groups. From the web survey data, we selected women who were in the same age range (over 50 years) as the patients (only one patient was 46 years, but the remaining patients were over 50), but the patients have a distribution peak at the older end of the age range, while the controls have a distribution peak at the younger end of the range. This is due to the fact that the web survey focused on younger women, so there is not much data available after the age of 50. As this is an important limitation of the study, we have added to the limitations of the revised manuscript that we could not match the ages of the patients and controls.

However, we found no significant correlation between age and body size distortion in the Controls. Moreover, the results of the sensitivity analysis were consistent with the results shown in the present study. Therefore, we believe that the controls used in this study are the data that could be used to compare body image and measurement data between NTM-LD patients and the general female population.

Table R2. Correlation between age and body size distortion in the controls.

| r | p-value |
| --- | --- |
| -0.058 | 0.543 |

n=111. Spearman’s correlation

Table R3. Anthropometric profiles and BSP in NTM-LD and age-matched Controls

|  | NTM-LD (n=81) | Controls (n=9) | p-value |
| --- | --- | --- | --- |
| Age (years) ^a^  Height (cm) ^a^  Body weight (kg) ^a^  Body mass index (kg/m^2^) ^a^  BSP ^b^  Underestimation of body size  No distortion  Overestimation of body size | 70.8 ± 8.6  153.7 ± 6.9  46.8 ± 7.2  19.8 ± 2.5  30 (37.0)  20 (24.7)  31 (38.3) | 68.0 ± 3.0  155.3 ± 5.1  53.6 ± 4.4  22.3 ± 1.8  5 (55.6)  2 (22.2)  2 (22.2) | 0.053  0.515  0.007  0.005  0.521 |

Mean ± standard deviation or n (%), ^a^ Unpaired t-test, ^b^ χ^2^ test

●*Revised Discussion (line 335-340):*

Third, the controls were younger than NTM-LD patients, so the two groups were not age-matched. However, there was no significant correlation between age and body size distortion in the controls. Moreover, the results of additional sensitivity analyses were consistent with the results of the present study. Therefore, we believe that controls were suitable for comparison of body image and measurement data with NTM-LD patients.

**4) Be consistent in the labelling of the OE vs Remaining….. maybe call these “non OE” or controls- see comment above.**

**Answer:** In response to your comment about the group name, "Remaining" has been changed to "Non-OE".

**5) Remove the “all” column… not sure what that adds, as the primary comparison is between cases and controls.**

**Answer:** Following your comment, the "all" column has been removed from Table 2.

**6) Lines 181-183: “impact of BMI (response variable) on age, etc”: this should be rephrased to “impact of age etc (these are the explanatory variables) on BMI (response variable).**

**Answer:** According to your comment and editor’s comment 7, following change has been made:

● *Revised Methods (line 182-185):*

“Multiple regression analysis was performed to evaluate the impact of age, duration of NTM-LD, percentage of weight loss from 20 years of age, normalized energy intake, appetite score, amount of physical activity, and body size distortion (independent variables) on BMI (dependent variable).

**7) Confusing to say “correlation between body size perception and BMI in NTM LD and controls”… .. since you have to have a control group to make these comparisons… suggest just saying “correlation between body size distortion and BMI“.**

**Answer:** Thank you for your kind suggestion. This sentence has been modified:

●*Original Results (line 204-207 and 215):*

“Correlations between body size distortion and BMI in NTM-LD and Controls”

↓

●*Revised Results (line 207 and 214):*

“Correlations between body size distortion and BMI**.**

**Comments by** **the Reviewer #2**

**The authors conducted a study about body size perception in patients with nontuberculous mycobacterial lung disease (NTM-LD). I agree that it is important to address the problem of nutritional status of patients with NTM-LD because low BMI is associated with poor prognosis. While I appreciate the effort of this study which included more than 80 female patients, there are some questions about the methodology of the study.**

**Answer:** We appreciate your thoughtful comments on our manuscript. We have revised our manuscript in response to all your comments.

**Major comments**

**1) Evaluation of body size perception**

**The main concern I have about this study is evaluation of body size perception (BSP). To assess patients' BSP, the authors used the Japanese version of body image scale they had developed. This scale was developed using the subjective responses and BMI from a healthy population (in which BMI was not evenly distributed), rather than using an image created from objective data of physical measurements. On this scale, the (mean) BMIs associated with low-numbered silhouettes seem to tend to shift to higher values.**

**When using this scale to analyze populations with very low BMI, doesn’t it affect the assessment of overestimation of one’s body size (OE)?**

**In fig1, there seems to be a certain number of NTM-LD patients with a BMI <16. Patients with a BMI <16 cannot be “underestimation (BSP <0)”, because the range starts at silhouette 1 = BMI 16.2 on this scale. This affected the distribution of dots in fig1. For this reason, BSP may have been more strongly associated with BMI in multiple regression analysis (table 4).**

**Isn't it difficult to accurately assess BSP without using an “objective” scale that extends the range of lower BMI?**

**Answer:** Thank you for your very important comment. As you mentioned, because the range starts at silhouette 1=BMI 16.2 kg/m^2^, which may have affected the results. So, we did a sensitivity analysis on Figure 1 and Table 4, excluding 10 patients with BMI less than 16.2 kg/m^2^. Sensitivity analysis shows the same results as follows：

Table R4. Correlations between body size distortion and BMI excluding patients with BMI less than 16.2.

| r | p-value |
| --- | --- |
| -0.392 | 0.001 |

n=71. Spearman’s correlation

Table R5. Multiple regression analyses with BMI as the response variable in NTM-LD patients with excluding patients BMI less than 16.2.

|  | β | 95% CI | VIF | p-value |
| --- | --- | --- | --- | --- |
| Age (years)  Duration of NTM-LD (month)  Percentage of weight loss from age 20 (%)  Energy intake (kcal/IBW1kg/day)  Appetite score (SNAQ-J)  Amount of physical activity (MET·hour /week)  Body size distortion | 0.127  -0.220  -0.488  0.115  0.090  0.182  -0.372 | -0.018 – 0.078  -0.015 – -0.001  -0.101 – 0.046  -0.026 – 0.101  -0.127 – 0.349  -0.001 – 0.020  -0.761 – -0.235 | 1.254  1.026  1.035  1.180  1.152  1.239  1.202 | 0.212  0.018  <0.001  0.244  0.354  0.073  <0.001 |

n=71.

　Moreover, the fact that some patients had lower BMI than the range of BMIs of the silhouettes we used should describe in the Limitations. We have also added a note about the desirability of developing and using the scales that expand the lower BMI range in the future research.

For, more objective measures (e.g., 3D composite silhouettes of body shape using actual measurements), it is difficult to obtain a large amount of data on thin Japanese females (both disease and healthy) immediately, so this will be an issue for the future. Thank you for your useful advice.

●*Revised Limitations (line 332- 335):*

“Second, 10 NTM-LD patients had lower BMI (> 16.2 kg/m2) than the range of BMI of the slimmest silhouette. Although the results excluding these 10 patients were consistent with the present results, further studies to develop and use the scales that expand the lower BMI range are needed.”

**2) BMI cutoffs for overestimation**

**Did the authors use the automatically calculated cutoff (Youden index?) in any of the "R" packages?**

**The BMI cutoff value for NTM-LD seems reasonable from the ROC (fig2). For the control, the BMI cutoff value from the ROC (fig2) seems to vary depending on the method. If a similar sensitivity (or specificity) to NTM-LD is chosen, the BMI cutoff value for the control is likely to be lower. In this study, it seems that there is no statistical meaning in examining the absolute value of the difference in the automatically calculated cutoff.**

**Answer:** Following your suggestion, Figure 2 has been removed from the revised manuscript. Also, in the Results section, we did not mention the cutoff values calculated from the ROC curves, but only indicated that the cutoff values were low or high.

●*Revised Results (line 211- 212):*

“BMI cutoff values in the NTM-LD (19.2 kg/m2) patients that overestimate their body size were lower than the controls (22.3 kg/m2).”

**Minor comments**

**1) line 31, line 70: Remove "pulmonary".**

**Answer:** In the Title, line 31, and line 70 (in the original manuscript), "pulmonary" has been removed. comment.

**2) line 133: Please provide details on how to convert BMI to silhouette number (actual silhouette).**

**Answer:** Based on your comment, the detail on how to convert BMI to silhouette number has been added.

●*Revised Methods (line 125- 134):*

“The 10 silhouettes had BMI ranges (mean ± standard error) calculated from data obtained from 444 Japanese women. The mean BMIs ranged from 16.2 (Silhouette 1) to 30.2 kg/m2 (Silhouette 10). Participants were asked to identify the silhouette that best represented their own body on the day of the interview or on the web survey (perceived silhouette). This BMI range was used to convert BMI to silhouette number [actual silhouette]. For those who deviated from the BMI range of each silhouette, the silhouette number of the closest BMI value was selected (n = 117) or those with a BMI lower than the BMI range of silhouette 1 (< 16.2 kg/m2) were assigned as silhouette 1 (n = 10, all NTM-LD patients).”
