# Supplementary material for: Association of body size distortion with low body mass index in female patients with nontuberculous mycobacterial lung disease
Source: PLoS One. 2023 Aug 22;18(8):e0290277. doi: 10.1371/journal.pone.0290277 (PMC10443841; doi:10.1371/journal.pone.0290277)
Supplement: Attachment — Submitted filename: Response to reviewers 2nd revision.docx [file pone.0290277.s005.docx]

**Point-by-point response to the Editor and Reviewer**

We are grateful to the editor and reviewer for important comments and useful suggestions. As indicated in the following responses, we have taken all these comments and suggestions into account in the revised version of our manuscript.

**Comments by the Editor**

**Major comments**

**1) Title: You misunderstood my comment, and you made an unwanted correction. The title should be expressed as “Association of body size distortion with low body mass index in female patients with nontuberculous mycobacterial lung disease”. I have never seen an expression such as “Association of [dependent variable] with [independent variable]” in scientific literature. To prevent readers’ confusion, you should express the “Association of [independent variable] with [dependent variable]” throughout the manuscript.**

**Answer:** We apologize for misunderstanding the editor's intentions and making unnecessary corrections. As you indicated, we have made the following corrections, the title and related sentences in throughout the manuscript.

● *Original Title:*

Association of low body mass index with body size distortion in female patients with non-tuberculous mycobacterial lung disease

↓

● *Revised Title:*

Association of **body size distortion** with **low body mass index** in female patients with nontuberculous mycobacterial lung disease

● *Original Running head:*

Low BMI and body size distortion in female nontuberculous mycobacterial lung disease patients

↓

● *Revised Running head:*

**Body size distortion** and **low BMI** in female nontuberculous mycobacterial lung disease patients

● *Original Abstract, Background (line 34-36):*

“To test this hypothesis, we examined whether the patients’ body mass index (BMI) was related to their body size perception (BSP), independent of age, disease duration, and nutritional factors.”

↓

● *Revised Abstract, Background (line 33-35):*

“The aim of this study was **to clarify whether patients’ body size perception (BSP) is associated with BMI independently of potential confounders**.”

● *Original Abstract, Methods (line 44-45):*

“Multiple regression analysis was performed to identify factors independently associated with patients' BMI.”

↓

● *Revised Abstract, Methods (line 43-46):*

　“Multiple regression analysis was performed **to clarify the association between BSP (independent variable) and BMI (dependent variable), adjusting for potential confounders, such as age, disease duration, and nutritional factors.**”

● *Original Introduction (line 97-98):*

“…patients' BMI was related to their BSP … “

↓

● *Revised Introduction* *(line 97-98):*

“…**patients' BSP (independent variable)** was related to **BMI (dependent variable)** …”

● *Original Statistical analysis (line 182-185)*

“Multiple regression analysis was performed to evaluate the impact of age, duration of NTM-LD, percentage of weight loss from 20 years of age, normalized energy intake, appetite score, amount of physical activity, and body size distortion (independent variables) on BMI (dependent variable).”

　　↓

● *Revised Statistical analysis (line 185-189)*

“Multiple regression analysis was performed to evaluate the impact of **body size distortion (independent variables) on BMI (dependent variable)** with adjustment for potential confounding factors (covariates), such as age, duration of NTM-LD, percentage of weight loss from 20 years of age, normalized energy intake, appetite score, and physical activity.”

● *Original Discussion (line 277)*

“to examine the association of low BMI with patients’ BSP”

↓

*● Revised Discussion (line 288)*

“to report the association of **BSP** with **low BMI**”

**2) Abstract; Page 2, lines 33-34: When you mention your hypothesis, you should use words, such as “would be” or “might be”. Your explanation “their thinness was associated with a misperception of body shape” is like a result (like a fact, not a hypothesis).**

**Answer:** Thanks for your advice on how to phrase the hypothesis. "Would be" or "might be" are usually used to predict experiments. However, according to academic writing textbooks, hypotheses should be written in the present tense, and modal verbs such as may, might, and could should not be used because they make the certainty unclear. Since the sentence "we hypothesized…" (lines 32-33) describes our hypothesis, not the prediction of the experiment, we do not think it is necessary to revise this sentence. We would appreciate your reconsideration.

**3) Abstract: Your descriptions regarding the multiple regression analysis are inconsistent in the Background and Method. In the Background, you mentioned that “To test this hypothesis, we examined whether the patients’ body mass index (BMI) was related to their body size perception (BSP), independent of age, disease duration, and nutritional factors.” However, in the Methods, you mentioned that “Multiple regression analysis was performed to identify factors independently associated with patients' BMI" which is totally different from the purpose of testing the above-mentioned hypothesis. Based on your descriptions in the Abstract (Background) and last paragraph of the Introduction section (lines 94-98), your purpose of the present study can be NOT to identify factors to be independently associated with BMI, but the current purpose is to clarify whether BSP is associated with BMI independently from potential confounding factors. Your inconsistent description in the Background and Methoｄs can confuse readers a lot. For testing your hypothesis, your description on the multiple regression analysis in the Method section should be expressed as “Multiple regression analysis was performed to clarify the association between BSP (independent variable) and BMI (dependent variable) with adjustment for potential confounders, such as age, disease duration, and nutritional factors”. Accordingly, the Results section should also be appropriately revised. For instance, it may be expressed as “multiple regression analysis revealed that overestimation of body size was associated with lower BMI independent of confounding factors, such as longer disease history nutritional factors, and longitudinal weight loss”.**

**Answer:** Thank you for pointing out the discrepancy in the description of entire manuscript. Also, thank you for your help with the example sentence. We have modified the revised manuscript according to your suggestions.

● *Original Abstract, Background (line 34-36):*

“To test this hypothesis, we examined whether the patients’ body mass index (BMI) was related to their body size perception (BSP), independent of age, disease duration, and nutritional factors.”

↓

● *Revised Abstract, Background (line 33-35):*

“The aim of this study was **to clarify whether patient’s body size perception (BSP) is associated with body mass index (BMI) independently of potential confounders**.”

● *Original Abstract, Methods (line 44-45):*

“Multiple regression analysis was performed to identify factors independently associated with patients' BMI.”

↓

● *Revised Abstract, Methods (line 43-46):*

　“Multiple regression analysis was performed **to clarify the association between BSP (independent variable) and BMI (dependent variable), adjusting for potential confounders, such as age, disease duration, and nutritional factors.**”

● *Original Abstract, Results (line 51-53):*

“Multiple regression analysis revealed that longer disease history, longitudinal weight loss, and overestimation of body size were all independently associated with lower BMI.”

↓

● *Revised Abstract, Results (line 52-55):*

“Multiple regression analysis revealed that **overestimation of body size was associated with lower BMI independent of confounders, such as longer disease history, longitudinal weight loss, and nutritional factors**.”

● *Original Results (line 248):*

　“To identify the factors related to patients’ BMI…”

↓

● *Revised Results (line 260-261):*

　“To identify the impact of **body size distortion on BMI with adjustment for potential confounders (covariates)** …”

● *Original Results (line 255-257):*

“These findings indicate that longer disease history, longitudinal weight loss, and overestimation of body size were all independently associated with low BMI.”

↓

● *Revised Results (line 266-268):*

“These findings indicate that **overestimation of body size was associated with lower BMI independent of confounders, such as longer disease history, longitudinal weight loss, and nutritional factors.”**

● *Original Discussion (line 272-274):*

“3) multiple regression analysis revealed that longer disease history, longitudinal weight loss, and overestimation of body size were all independently associated with lower BMI.”

↓

● *Revised Discussion (line 283-285):*

“3) multiple regression analysis revealed that **overestimation of body size was associated with lower BMI independent of confounders, such as longer disease history, longitudinal weight loss, and nutritional factors**.”

● *Original Discussion (line 304-307):*

In the multiple regression analysis, duration of NTM-LD, percentage weight loss after 20 years of age, and body size overestimation were identified as factors significantly associated with the lower BMI in NTM-LD patients independent of age, appetite, normalized energy intake, and amount of physical activity.

↓

● *Revised Discussion (line 321-324):*

In the multiple regression analysis, body size overestimation was identified as an independent variable significantly associated with the lower BMI in the patients independent of the following confounders: age, duration of the disease, longitudinal weight loss, normalized energy intake, appetite, and amount of physical activity.

● *Original Discussion (line 307-315):*

Based on above collections, the following sentences have been deleted.

“The association between longer disease duration and lower BMI supports previous studies that reported that weight loss may occur as a symptom or as a result of treatment in patients with NTM-LD [30, 31]. The association with long-term weight loss can be explained by the wasting associated with the disease, which leads to a relative shortage of energy intake or a negative energy balance, with the result that the higher the long-term weight loss experienced by the patients, the lower their BMI. In the present study, patients' overestimation of their own body size was associated with a lower BMI independent of other factors.”

● *Original Discussion (line 323-326):*

“Thus, the assessment of long-term weight change, BSP, and its distortion may aid in the nutritional management of female patients with NTM-LD, prevent weight loss, and thereby exert a beneficial effect on treatment outcomes.”

↓	“long-term weight change” has been deleted.

● *Revised Discussion (line 333-336):*

“However, the results of this study suggest that **the assessment of BSP** and its distortion may help in the nutritional management of female patients with NTM-LD to prevent weight loss, and thereby exert a beneficial effect on treatment outcomes.”

**4) Page 5, lines 96-98: If you mentioned “BSP was related to BMI (independent variable comes first, followed by the dependent variable)”, it may not cause readers’ confusion. You should add explanations with parentheses like “patients' BMI** **(dependent variable) was related to their BSP** **(independent variable)” to prevent readers’ confusion.**

**Answer:** Following your comments, explanations have been added in the revised manuscript~~.~~

● *Original Introduction (line 97):*

“…patients' BMI was related to their BSP … “

↓

● *Revised Introduction* *(line 97-98):*

“…**patients' BSP (independent variable)** was related to **BMI (dependent variable)** …”

**5) Page 9, lines 182-185: As mentioned above, based on your descriptions in the Abstract (Background) and last paragraph of the Introduction section (lines 94-98), the purpose of the present study is to clarify whether BSP is associated with BMI independently from potential confounding factors. Thus, this explanation of the multiple regression analysis is wrong. According to the purpose of the current study, you should mention that “Multiple regression analysis was performed** **to evaluate the impact of body size distortion (independent variables) on BMI (dependent variable)** **with adjustment for potential confounding factors (covariates), such as age, duration of NTM-LD, percentage of weight loss from 20 years of age, normalized energy intake, appetite score, and physical activity.**

**Answer:** Thank you for your suggestion, we have made the following correction.

● *Original Method (line 182-185):*

*“*Multiple regression analysis was performed to evaluate the impact of age, duration of NTM-LD, percentage of weight loss from 20 years of age, normalized energy intake, appetite score, amount of physical activity, and body size distortion (independent variables) on BMI (dependent variable).”

↓

● *Revised Method (line 185-189):*

*“*Multiple regression analysis was performed to evaluate the impact of **body size distortion (independent variables) on BMI (dependent variable) with adjustment for potential confounding factors (covariates), such as age, duration of NTM-LD, percentage of weight loss from 20 years of age, normalized energy intake, appetite score, and physical activity**.”

**6) Page 14, Table 4: It is OK to show the results (β values, their CI, and P values) of confounding factors such as age, but the results (β values, their CI, and P values) for the most important variable, body size distortion, should be shown in the first top line (not the last or bottom line) of Table 4. The title may be revised to “Association of BSP with BMI in NTM-LD patients”.**

**Answer:** Based on your suggestions, we have changed the title of Table 4, and the order of the variables have been modified, as follows:

● *Original title of Table 4 (line 259-260):*

*“*Multiple regression analyses with BMI as the dependent variable in NTM-LD patients”

↓

● *Revised title of Table 4 (line 271):*

*“*Association of BSP with BMI in NTM-LD patients.”

● *Revised Table 4*

The most important result of BSP has been moved to the top of the Table 4.

**7) Page 9, lines 185-187: You misunderstood my previous comment on the confounding or mediating factors, and you made an unwanted correction. Whether a certain variable is treated as a confounding factor or mediating factor should be determined before performing statistical analysis, based on the authors’ interest (and authors’ hypothesis), but not based on the results of the mediation analysis. For instance, if you are interested in testing a hypothesis that the effect of BSP (independent variable) on BMI (dependent variable) would be mediated by total energy intake, it is appropriate to treat the variable (total energy intake) as a potential mediating factor. Thus, in this case, it is also appropriate to perform a mediation analysis. Since it was unclear to me whether you are interested in “the mediation” in the previous version of the manuscript, I thus asked whether you are treating variables as confounding factors or mediating factors. Based on the present version of the manuscript, it appears that you are not interested in any mediation, and do not have any hypothesis regarding mediation. Thus, you should not perform any mediation analysis in the present study. You should delete all the descriptions related to mediation analysis from the manuscript.**

**The results and discussion sections should also be carefully revised according to the new revisions regarding the statistical analyses.**

**Answer:** We apologize for misunderstanding your comments. We have eliminated all the descriptions related to mediation analysis from the revised manuscript. We have also carefully reviewed the revised manuscript, but we did not find the description of the mediation analysis in the revised Results and the Discussion sections.

● *Original Methods (line 185-187):*

*“*We obtained an average causal mediation effect (ACME), an average direct effect, and a total effect using the R mediation package.”

↓

● *Revised Methods*

Above sentence has been eliminated.

● *Original Results (line 253-255):*

*“*Causal mediation analysis showed that weight loss from age 20 years did not mediate −0.146 (95% CI, −0.388 to 0.050) BSP in BMI (p = 0.140 for ACME). Other factors also did not mediate the effect of BMI.”

↓

● *Revised Results*

Above sentence has been eliminated.

**8) -1 Overall, I agree with Reviewer 2’s comments.**

**I understand that you can't do anything about having used the Japanese version of the body image scale that you own developed. However, you may be able to emphasize the most important limitations (pointed out by Reviewer 2) more clearly, meticulously, and courteously, by making one new paragraph (separately from the limitation paragraph) in the Discussion section.**

**Answer:** Thank you for your suggestion. We have added a new paragraph in the Discussion regarding the reasons and limitations of using the Japanese version of the Body Image Scale.

● *Revised Discussion (line 339-351)*

“In this study, we used the J-BIS [19], which we developed and confirmed its reliability and validity in the Japanese female population, to assess the subjects' BSP. Although there are several studies that have assessed body size perception using the BIS [27, 28, 36], there are some problems as follows: First, the J-BIS was selected based on the subjective evaluation of the participants. As seen in the present results, even healthy controls, especially women with low BMI, may have biased their perception of body size. Moreover, the J-BIS was developed for Japanese women and published in Japanese journal, making it difficult for foreign readers to confirm its reliability and validity or to use the J-BIS. Furthermore, the NTM-LD patients were thinner than the healthy controls, and 10 NTM-LD patients had BMI (> 16.2 kg/m^2^) lower than the BMI range of the slimmest silhouette 1. Although the results excluding these 10 patients were in close agreement with the present results, it is necessary to develop a new BIS that can adequately cover slimmer subjects in the future.”

**8)-2 I understand that you performed the sensitivity analyses with the exclusion of patients whose BMI < 16.2, as a last resort (Kunikunosaku in Japanese). However, I could not find any descriptions regarding the sensitivity analyses (in the Methods or Results sections, or Tables) in the revised manuscript.**

**Answer:** According to your comments, the descriptions regarding the sensitivity analyses (in the Methods or Results sections) have been added in the revised manuscript.

● *Revised Methods (line 189-191)*

“We conducted a sensitivity analysis after excluding participants with a BMI of less than 16.2 kg/m^2^ (all NTM-LD patients).”

● *Revised Results (line 216-218)*

“Similar results (*r* = -0.392, *p* = 0.001, *Spearman'*s correlation) were obtained in a sensitivity analysis excluding NTM-LD patients with BMI of less than 16.2 kg/m^2^ (*n* = 71).”

● *Revised Results (line 246-249)*

“Sensitivity analysis excluding NTM-LD patients with BMI of less than 16.2 kg/m^2^ yielded similar results for anthropometric data, but for nutritional intake, the significant differences in energy and protein intake disappeared (S2 Table). “

● *Revised Results (line 268-269)*

“Sensitivity analysis excluding NTM-LD patients with BMI of less than 16.2 kg/m^2^ showed similar results (S3 Table). “

**8)-3 Your revision according to the precious comments from Reviewer 2 has not been incorporated into the revised manuscript. I suggest you show the results of sensitivity analyses as supplemental files.**

**Answer:** Thank you for your suggestion. The results of the sensitivity analysis have been shown in the Supplementary tables (S1-S3 Table).

**8)-4 A thorough discussion on the results of sensitivity analyses is needed.**

**Answer:** According to your comment, follow sentences have been added in the revised Discussion.

● *Revised Discussion (line 310-318)*

“However, the results of the sensitivity analysis excluding patients with a BMI of less than 16.2 kg/m2 showed that the significant differences in energy and protein intake between the OE and non-OE groups disappeared, leaving a significant difference only in long-term weight loss. Given the body size distortion and BMI of the healthy controls shown in Fig 1, the frequency of patients with distortion may change significantly if a scale is developed using an expanded control group that includes thinner women. Further studies with the newly developed body image scale are needed to support the present results.”

**8)-5 Unfortunately, your response to the Reviewer 2’s comment on the BMI cutoff values is also insufficient.**

**Answer:** We have taken seriously your suggestion that our response to the previous reviewer 2 was inadequate. The descriptions of the BMI cutoff value (in the Methods or Results sections) have been added in the revised manuscript. Again, our response is as follows:

● *Revised Method (line 179-185)*

The follow section has been added.

“The optimal area under the curve (AUC), sensitivity, and specificity were calculated using a receiver operating characteristic (ROC) curve analysis. The Youden index was used to determine the optimal cut-off value for the target value. Diagnostic accuracy using the area under the ROC curve was defined as follows; < 0.5 (test not useful), 0.5-0.6 (poor), 0.6-0.7 (sufficient), 0.7-0.8 (good), 0.8-0.9 (very good), and 0.9-1.0 (excellent) using the R software package (version 4.0.0 for Windows, R Core Team, Vienna, Austria).”

*● Original Result (line 211-212):*

BMI cutoff values in the NTM-LD (19.2 kg/m^2^) patients that overestimate their body size were lower than the controls (22.3 kg/m^2^).

↓

● *Revised Result (line 218-220)*

“BMI cutoff values in the NTM-LD (19.2 kg/m^2^, **Sensitivity = 0.800, Specificity = 0.677**) patients that overestimate their body size were lower than the controls (22.3 kg/m^2^, **Sensitivity = 0.500, Specificity = 0.857**).”

*● Original Discussion (line 286-290):*

“A high rate of overestimation was observed in the female patients in this study, even though their average age was over 70 years. Such overestimation may be caused by factors that are different from those in younger women. Further studies are needed.”

↓

● *Revised Discussion (line 297-302)*

“… **as many as 40% of** overestimation was observed in the female patients in this study, even though their average age was over 70 years. **In addition, BMI cutoff values in the patients that overestimate their body size were approximately 3 kg/m^2^ lower than the controls**. Such overestimation may be caused by factors that are different from those in younger women. Further studies will be necessary for clarification of this issue.”

**Comments by the Reviewer #2**

**Major comments**

**1)-1 Evaluation of body size perception**

**I still think that it is important to define a standard body image to evaluate body size perception. Unfortunately, readers outside Japan will not be able to confirm the validity of the scale used in this study.**

**Answer:** According to your comment and editor’s comment 8-1, a new paragraph was added to the revised Discussion regarding the limitations of J-BIS being developed for Japanese women and published in Japanese paper.

● *Revised Discussion (line 345-347)*

The follow section has been added.

“… Moreover, the J-BIS has been developed for Japanese women and published in Japanese paper, making it difficult for foreign readers to confirm its reliability and validity or to use the J-BIS.”

**1)-2 As the authors describe, even healthy controls (especially with low BMI) can have body size distortion. Therefore, in order to assess BSD properly, it seems necessary to use a scale developed by the objective indicators rather than a scale developed by the subjective judgment of controls. The use of authors’ scale seems to be a major limitation of this study when assessing “overestimation of body size”. Shouldn't the authors clearly state the issue?**

**Answer:** Following your comment and the editor’s comment (8-1), a new paragraph has been ~~was~~ added to the revised Discussion to describe the limitations of using a scale developed on the basis of subjective judgment (evaluation).

● *Revised Discussion (line 342-345)*

The follow section has been added.

“… First, the J-BIS was selected based on the subjective evaluation of the participants. As seen in the present results, even healthy controls, especially women with low BMI, may have biased their perception of body size.”

**1)-3 In this paper, the authors seem to overemphasize the frequency of patients with OE (~40%). Considering body size distortion and BMI in the controls shown in fig 1, the frequency of patients with distortion could vary significantly if a scale is developed using the controls in this study (or the expanded control set).**

**Answer:** We appreciate your thoughtful comments on our manuscript. We have modified the revised manuscript according to your suggestions.

● *Revised Discussion (line 310-318)*

The follow section has been added.

“However, the results of the sensitivity analysis excluding patients with a BMI of less than 16.2 kg/m^2^ showed that the significant differences in energy and protein intake between the OE and non-OE groups disappeared, leaving a significant difference only in long-term weight loss. Given the body size distortion and BMI of the healthy controls shown in Fig 1, the frequency of patients with distortion may change significantly if a scale is developed using an expanded control group that includes thinner women. Further studies with the newly developed body image scale are needed to support the present results.”

**1)-4 In addition, though there is no significant difference between the frequency of OE in the patients with NTM-LD and the controls, the authors seem to emphasize the higher frequency of OE in the patients (line 195, line 277...).**

**As authors hypothesized (line 90),** **the female patients with** **NTM-LD have body size distortion similar to that of the general adult female population.** **In this study, a strong association between low BMI and OE is shown in the patients as well as in the controls.**

**Answer:** We agree with your comment. We have modified following sentences:

*● Original Results (line 195-197):*

　“… however, NTM-LD had a higher proportion of respondents who overestimated their body size than controls…”

　　↓

● *Revised Results (line 199-200)*

　“… however, NTM-LD had **overestimated their body size similar to that of the controls** …”

*● Original Discussion (line 268-270):*

“1) female patients with NTM-LD were thinner than the control women from the general population; however, about 40% of the patients overestimated their body size …”

　　↓

● *Revised Discussion (line 280-281)*

“1) **a strong association between OE and low BMI is shown in the patients as well as in the controls** …”

*● Original Discussion (line 277-279):*

“About 40% of the present patients overestimated their body size, which was higher than the about 30% of control women in the general population.”

　 ↓

● *Revised Discussion (line 288-291)*

“In this study, **as in the general female population, approximately 40% of patients overestimated their body size, and a strong association was found between body size overestimation and low BMI.**”

*● Original Discussion (line 286-288):*

“A high rate of overestimation was observed in the female patients in this study, even though their average age was over 70 years.”

　 ↓

● *Revised Discussion (line 297-299)*

…**as many as 40%** **of** overestimation was observed in the female patients in this study, even though their average age was over 70 years.

**2) Sensitivity analysis**

**Authors performed a sensitivity analysis on Figure 1 and Table 4, excluding 10 patients with BMI less than 16.2 kg/m2. Sensitivity analysis showed that the patients (r=-0.392) had a weaker correlation between BMI and BSP than the controls (r=-0.497).**

**How about the frequency of “overestimation of body size” in table 1 and “Nutritional intake” in table 3 when excluding the patients with BMI less than 16.2 kg/m2?**

**Answer:** Thank you for your comments, we have created new supplement tables for “the frequency of body size overestimation” in S1 Table and "Nutritional Intake" in S2 Table when patients with BMI less than 16.2 kg/m^2^ has been excluded. According to your comment and editor’s comment (8-4), we have included the results of the sensitivity analysis as a supplemental file (S1-S3 Tables) and added the following to the revised Methods and Results section.

**S1 Table.** Anthropometric profiles BSP in NTM-LD and Controls with excluding patients BMI less than 16.2 kg/m^2^

|  | NTM-LD  (*n* = 71) | Controls  (*n* = 111) | *p* value ^a^ |
| --- | --- | --- | --- |
| Age (years)  Height (cm)  Body weight (kg)  Body mass index (kg/m^2^)  BSP ^b^  Underestimation of body size  No distortion  Overestimation of body size | 70.5 ± 8.9  153.8 ± 6.8  48.2 ± 6.4  55.8 ± 8.5  30 (42.3)  17 (23.9)  24 (33.8) | 57.1 ± 5.2  157.9 ± 5.3  55.8 ± 8.5  22.4 ± 3.2  44 (39.6)  36 (32.4)  31 (27.9) | **< 0.001**  **< 0.001**  **< 0.001**  **< 0.001**  0.441 |

Mean ± standard deviation or *n* (%), ^a^ Unpaired t-test, ^b^ *χ^2^* test

BSP, body size perception; NTM-LD, nontuberculous mycobacterial lung disease

Patients were divided into underestimation of body size, no distortion, and overestimation of body size based on the difference between their perceived and actual silhouettes.

**S2 Table.** Comparison of anthropometric and nutritional characteristics between the patient groups with excluding BMI less than 16.2 kg/m^2^

|  | OE  (*n* = 24) | Non-OE  (*n* = 47) | *p* value |
| --- | --- | --- | --- |
| Anthropometric data  Height (cm)  Body weight (kg)  Body mass index (kg/m^2^)  Fat mass (kg)  Percentage of body fat (%)  Skeletal muscle mass (kg)  Weight loss from age 20 (kg)  Percentage of weight loss from age 20 (%)  Weight loss from maximum body weight (kg)  Percentage of weight loss from maximum body weight (%)  Nutritional intake  Energy (kcal/day)  Energy (kcal/IBW1kg/day) ^a^  Protein (g/day)  Protein (g/IBW1kg/day) ^a^  Fat (g/day)  Fat (g/IBW1kg/day) ^a^  Carbohydrate (g/day)  Carbohydrate (g/IBW1kg/day) ^a^  Appetite score (SNAQ-J)  Amount of physical activity (MET·hour/week)  Duration of sitting time (hour/day)  Blood biochemistry  Albumin (g/dL)  Transthyretin (mg/dL) ^b^  Transferrin (mg/dL)  Retinol-binding protein (mg/dL) ^b^  Total cholesterol (mg/dL)  Hemoglobin (g/dL)  Lymphocyte count (×10^2^/μL) | 157.7 ± 6.6  46.2 ± 4.9  19.0 ± 1.7  11.4 ± 3.9  24.2 ± 6.5  18.2 ± 1.9  3.5 ± 6.9  6.4 ± 12.7  8.6 ± 6.6  15.2 ± 10.6  1658 ± 323  31.1 ± 6.4  61.6 ± 18.4  1.2 ± 0.4  49.0 ± 16.5  0.9 ± 0.3  231.5 ± 49.8  4.3 ± 0.9  14.9 ± 1.9  26.8 ± 34.6  4.6 ± 2.9  4.4 ± 0.3  19.4 ± 4.7  219.3 ± 45.9  2.4 ± 0.6  224.3 ± 29.2  13.0 ± 0.8  12.8 ± 4.7 | 152.8 ± 6.7  49.3 ± 6.8  21.0 ± 2.0  14.9 ± 4.1  29.9 ± 5.6  18.0 ± 2.5  -0.6 ± 6.5  -2.0 ± 13.7  6.1 ± 5.5  10.7 ± 8.4  1728 ± 352  33.6 ± 6.3  68.3 ± 17.6  1.3 ± 0.3  54.1 ± 18.0  1.0 ± 0.3  233.8 ± 61.0  4.6 ± 1.1  30.9 ± 42.5  30.9 ± 42.5  4.9 ± 2.0  4.4 ± 0.3  20.4 ± 4.3  224.8 ± 34.1  2.6 ± 0.5  226.5 ± 38.1  13.2 ± 1.3  14.3 ± 3.6 | 0.089  **0.034**  **< 0.001**  **0.001**  **< 0.001**  0.685  **0.015**  **0.017**  0.094  0.055  0.417  0.126  0.138  0.051  0.253  0.146  0.869  0.428  0.267  0.686  0.627  0.543  0.348  0.568  0.257  0.805  0.675  0.136 |

Mean ± standard deviation, Unpaired t-test (OE vs. Non-OE).

^a^ Normalized using ideal body weight (BMI = 22 kg/m^2^).

^b^ Data (transthyretin and retinol-binding protein) of one patient in the Non-OE group could not be obtained.

NTM-LD, nontuberculous mycobacterial lung disease; IBW, ideal body weight; SNAQ-J, Japanese version of the Simplified Nutritional Appetite Questionnaire

**3) BMI cutoffs for overestimation**

**Revised Results (line 211- 212):**

**“BMI cutoff values in the NTM-LD (19.2 kg/m2) patients that overestimate their body size were lower than the controls (22.3 kg/m^2^).”**

**Again, how did the authors calculate BMI cutoff values?**

**Without knowing the sensitivity and specificity for each cutoff value, it is difficult to compare values.**

**Answer:** We apologize for the inadequate response. According to your comment and editor’s comment (8-5), we have added the sentences regarding sensitivity and specificity for each cutoff value in the Method and Result sections.

*● Revised Method (line 179-185)*

“The optimal area under the curve (AUC), sensitivity, and specificity were calculated using a receiver operating characteristic (ROC) curve analysis. The Youden index was used to determine the optimal cut-off value for the target value. Diagnostic accuracy using the area under the ROC curve was defined as follows; < 0.5 (test not useful), 0.5-0.6 (poor), 0.6-0.7 (sufficient), 0.7-0.8 (good), 0.8-0.9 (very good), and 0.9-1.0 (excellent) using the R software package (version 4.0.0 for Windows, R Core Team, Vienna, Austria).”

*● Original Result (line 211-212):*

“BMI cutoff values in the NTM-LD (19.2 kg/m^2^) patients that overestimate their body size were lower than the controls (22.3 kg/m^2^).”

↓

*● Revised Result (line 218-220)*

“BMI cutoff values in the NTM-LD (19.2 kg/m^2^, **Sensitivity = 0.800, Specificity = 0.677**) patients that overestimate their body size were lower than the controls (22.3 kg/m^2^, **Sensitivity = 0.500, Specificity = 0.857**).”

**Other changes**

Besides responses to the editor and the reviewer comments, we have made the following changes in the revised manuscript.

*● Original Discussion (line 282-286):*

“The lower incidence of overestimation in the general female population in the present study than in the early studies may be due to the younger age of the participants (> 17 years [27−29] vs. > 50 years), as younger age is more likely to lead to overestimation of body size in adult women [18]”.

　↓

*● Revised Discussion (line 294-297):*

“The lower incidence of overestimation in the general female population in the present study than in the early studies may be due to the **older** age of the participants **(> 50 years vs. > 17 years [27−29]**), as younger age is more likely to lead to overestimation of body size in women [18]”.

*● Original Discussion (line 288-290):*

“Such overestimation may be caused by factor 2) examined s that are different from those in younger women. Further studies are needed.”

　　↓

*● Revised Discussion (line 300-302):*

“Such overestimation may be caused by factors different from those in younger women. Further studies **will be necessary for clarification of this issue**.”
