# Supplementary material for: Association of body size distortion with low body mass index in female patients with nontuberculous mycobacterial lung disease
Source: PLoS One. 2023 Aug 22;18(8):e0290277. doi: 10.1371/journal.pone.0290277 (PMC10443841; doi:10.1371/journal.pone.0290277)
Supplement: Attachment — Submitted filename: Response to reviewers 3rd revision.docx [file pone.0290277.s006.docx]

**Point-by-point response to the Editor and Reviewer**

We thank the Editor and the Reviewer for the valuable comments which surely help to improve the manuscript. As indicated in the following responses, we have taken all these comments and suggestions into account in the revised version of our manuscript.

**Journal Requirements**

**Please review your reference list to ensure that it is complete and correct. If you have cited papers that have been retracted, please include the rationale for doing so in the manuscript text, or remove these references and replace them with relevant current references. Any changes to the reference list should be mentioned in the rebuttal letter that accompanies your revised manuscript. If you need to cite a retracted article, indicate the article’s retracted status in the References list and also include a citation and full reference for the retraction notice.**

**Answer:** We apologize for any insufficiencies in our previous response. The details of the reference changes (two removals and one addition) are as follows.

● Due to the change in the Discussion section (removal of the sentence containing ref. 30 and ref. 31), these references have been removed since the 2nd revision.

● Due to the addition of the limitation section sentence, ref. 36 has been added since the 2nd revision.

● As a result of the above changes, the numbers in references 32-37 have been moved up by two and the number in reference 38 has been moved up by one (these are not new additions).

● We have verified that all of the references in the revised manuscript (the most recent) are correct.

**Comments by the Editor**

**1) To be consistent with Table 1, a superscript "a" of p value should be moved to the variables (age, height, body weight, and body mass index) in S1 Table.**

**Answer:** Thank you for pointing out the discrepancy in the description of Table 1. We have modified the revised S1 Table according to your suggestions.

● *Revised S1 Table:*

**Comments by the Reviewer #2**

**1)** **BMI cutoffs for overestimation**

**line 184: “Diagnostic accuracy using the area under the ROC curve was defined as follows; < 0.5 (test not useful), 0.5-0.6 (poor), 0.6-0.7 (sufficient), 0.7-0.8 (good), 0.8-0.9 (very good), and 0.9-1.0 (excellent) using the R software package.”**

**The values of area under the ROC curve (AUC) are not described in the results section of the revised manuscript.**

**Answer:** We apologize for the inadequate answer. We have added the AUC sentences for each cutoff value in the Results section.

● *Original Results (line 218-220):*

“BMI cutoff values in the NTM-LD (19.2 kg/m^2^, Sensitivity = 0.800, Specificity = 0.677) patients that overestimate their body size were lower than the controls (22.3 kg/m^2^, Sensitivity = 0.500, Specificity = 0.857).”

↓

● *Revised Results (line 218-221):*

“BMI cut-off values in the NTM-LD patients (19.2 kg/m^2^, **AUC = 0.784, Sensitivity = 0.677, Specificity = 0.800**) **overestimating** their body size were lower than **in** the controls (22.3 kg/m^2^, **AUC = 0.708, Sensitivity = 0.857, Specificity = 0.500**), **although the sensitivity was lower in the patients**.”

**2) line 224: “BMI cutoff values in the NTM-LD (19.2 kg/m^2^, Sensitivity = 0.800, Specificity = 0.677) patients that overestimate their body size were lower than the controls (22.3 kg/m^2,^ Sensitivity = 0.500, Specificity = 0.857).”**

**Looking at fig2 of the first version of the paper, it seems that sensitivity and specificity are mixed up in the revised paper. (Please check for any mistakes. Sensitivity = 0.677, Specificity = 0.800 for NTM-LD? and Sensitivity = 0.857, Specificity = 0.500 for the controls?)**

**If you mention that the cutoff value is lower in the NTM-LD patients, you should also mention that** **the sensitivity is lower in the patients. I think that there is little significance in comparing cut-off values with different sensitivities.**

**Answer:** Thank you for your careful confirmation. We also apologize for our inadequate corrections. As you pointed out, we have made the following corrections.

● *Original Results (line 218-220):*

“BMI cutoff values in the NTM-LD (19.2 kg/m^2^, Sensitivity = 0.800, Specificity = 0.677) patients that overestimate their body size were lower than the controls (22.3 kg/m^2^, Sensitivity = 0.500, Specificity = 0.857).”

↓

● *Revised Results (line 218-221):*

“BMI cut-off values in the NTM-LD patients (19.2 kg/m^2^, **AUC = 0.784, Sensitivity = 0.677, Specificity = 0.800**) **overestimating** their body size were lower than **in** the controls (22.3 kg/m^2^, **AUC = 0.708, Sensitivity = 0.857, Specificity = 0.500**), **although the sensitivity was lower in the patients**.”

● *Original Discussion (line 299-300):*

“In addition, BMI cutoff values in the patients that overestimate their body size were approximately 3 kg/m^2^ lower than the controls.”

↓

● *Revised Discussion*

Above sentence has been eliminated.
