# Supplementary material for: Association of body size distortion with low body mass index in female patients with nontuberculous mycobacterial lung disease
Source: PLoS One. 2023 Aug 22;18(8):e0290277. doi: 10.1371/journal.pone.0290277 (PMC10443841; doi:10.1371/journal.pone.0290277)
Supplement: Attachment — Submitted filename: Response to reviewers 4th revision.docx [file pone.0290277.s007.docx]

**Point-by-point response to the Editor and Reviewer**

We thank the editor for the valuable comments which certainly help to improve the manuscript. As indicated in the following responses, we have taken all these comments and suggestions into account in the revised version of our manuscript.

**Journal Requirements**

**Please review your reference list to ensure that it is complete and correct. If you have cited papers that have been retracted, please include the rationale for doing so in the manuscript text, or remove these references and replace them with relevant current references. Any changes to the reference list should be mentioned in the rebuttal letter that accompanies your revised manuscript. If you need to cite a retracted article, indicate the article’s retracted status in the References list and also include a citation and full reference for the retraction notice.**

**Answer:** We have verified that all of the references in the revised (latest) manuscript are correct.

**Comments by the Editor**

**1) Page 9, lines 179-185: I have recognized that the explanation of ROC curve analysis is insufficient. Readers will be confused a lot, because even the purpose of the ROC curve analysis is not clearly described, and the methodology of ROC curve analysis is inappropriately described in the Methods section. Please consider the following descriptions: The receiver operating characteristic (ROC) curves were drawn to assess the prediction performance regarding the BMI and body size overestimation (OE) in the NTM-LD and control groups, and their area under the curves (AUC) were calculated using ROC curves. To compare the optimal cut-off values of BMI to best predict the body size OE between the two groups, the optimal cut-off values were calculated by Youden index, and the sensitivity and specificity under the optimal cut-off points in the ROC curves were obtained.**

**Although these suggested descriptions may not be correct, you can use them as a reference for your appropriate revision. Again, please explain the purpose and method of the ROC curve analysis more clearly and accurately for readers.**

**Answer:** Thank you for your suggestion as well as providing sample sentences. We apologize for the incomplete revision of our manuscript regarding ROC curve analysis. I and co-authors have fully considered your suggestions and made corrections. We hope that the change we have made are satisfactory to you.

● *Original Methods (line 179-185):*

“The optimal area under the curve (AUC), sensitivity, and specificity were calculated using a receiver operating characteristic (ROC) curve analysis. The Youden index was used to determine the optimal cut-off value for the target value. Diagnostic accuracy using the area under the ROC curve was defined as follows; < 0.5 (test not useful), 0.5-0.6 (poor), 0.6-0.7 (sufficient), 0.7-0.8 (good), 0.8-0.9 (very good), and 0.9-1.0 (excellent) using the R software package (version 4.0.0 for Windows, R Core Team, Vienna, Austria).”

↓

● *Revised Methods (line 179-187):*

“The receiver operating characteristic (ROC) curves were drawn to assess the prediction performance regarding the BMI and body size overestimation in the NTM-LD and control groups, and their area under the curves (AUC) were calculated using ROC curves. To compare the optimal cut-off values of BMI to best predict the body size overestimation between the two groups, the optimal cut-off values were calculated by Youden index, and the sensitivity and specificity under the optimal cut-off points in the ROC curves were obtained. The ROC analyses were performed using the R software package (version 4.0.0 for Windows, R Core Team, Vienna, Austria).”

**2) Page 11, line 219: This sentence may also not be correct. Consider the following sentence “The optimal BMI cut-off values in the NTM-LD patients (19.2 kg/m^2^, AUC = 0.784, Sensitivity = 0.677, Specificity = 0.800) best predicting the overestimation of body size were ……”.**

**Answer:** Thanks for your advice. Based on your suggestion, the following correction has been made.

● *Original Results (line 218-221):*

　“BMI cut-off values in the NTM-LD patients (19.2 kg/m^2^, AUC = 0.784, Sensitivity = 0.677, Specificity = 0.800) overestimating their body size were lower than in the controls (22.3 kg/m^2^, AUC = 0.708, Sensitivity = 0.857, Specificity = 0.500), although the sensitivity was lower in the patients.”

↓

● *Revised Results (line 220-224):*

　“The optimal BMI cut-off values in the NTM-LD patients (19.2 kg/m^2^, AUC = 0.784, Sensitivity = 0.677, Specificity = 0.800) best predicting the overestimation of body size were lower than in the controls (22.3 kg/m^2^, AUC = 0.708, Sensitivity = 0.857, Specificity = 0.500), although the sensitivity was lower in the patients.”
